# Comparative Diagnostic Value of Computed Tomography Lung and Bone Window Settings for the Detection of Nasal Foreign Bodies in 47 Dogs Presented to Two UK Referral Hospitals (2015–2023)

**DOI:** 10.3390/ani15182684

**Published:** 2025-09-13

**Authors:** Nicoletta Fantaconi, Andrew T. Parry, Jose Labrador, Luis Alejandro Pérez López, Petra Agthe

**Affiliations:** 1 Department of Diagnostic Imaging, Anderson Moores Veterinary Specialists, Winchester SO21 2LL, UK; petra.agthe@scvetspecialists.co.uk; 2 Antech Imaging Services, Fountain Valley, CA 92708, USA; 3 Department of Diagnostic Imaging, Veterinary Emergency and Specialty-VES Hospital, Singapore 297824, Singapore; 4 Department of Diagnostic Imaging, Davies Veterinary Specialists, Hitchin SG5 3HR, UK

**Keywords:** bone window (BW), computed tomography (CT), dog, foreign bodies, lung window (LW), nasal cavities, nasal diseases, nasal foreign bodies

## Abstract

Confident diagnosis of nasal foreign bodies (FBs) with computed tomography (CT) is challenging. Plant material FBs may be inconspicuous depending on size and attenuation and may be obscured by secondary nasal changes such as accumulation of mucous. The authors anecdotally observed that the lung window (LW) might improve visualization of some nasal FBs. The aim of this retrospective, multicentre study was to assess the diagnostic utility and interobserver variability of the LW in the diagnosis of nasal FBs. We hypothesized that use of the LW improves detection rate of nasal FBs compared to the bone window (BW), and that interobserver agreement is strong. Computed tomography examinations of 47 dogs with an endoscopically confirmed nasal foreign body (FB) were included, and each study was reviewed independently by two board certified radiologists, resulting in a total of 94 assessments. Pre-contrast CT series were reviewed in the BW and LW. The reviewers were blinded to the final diagnosis and were asked to evaluate the CT studies for presence or absence of a convincing nasal FB. Reviewers confidently detected a nasal FB on 19/94 (20%) assessments in the BW and 20/94 (21%) in the LW, the majority of the FBs were elongated in shape (30%) and were visible in the rostral and mid-portion of the nasal cavity. The interobserver agreement was moderate in the BW (*k* < 0.53) and in the LW (*k* < 0.49). Our findings do not support our main hypothesis that the use of the LW significantly increases diagnostic accuracy for the identification of nasal FBs in dogs. However, as the LW enabled correct diagnosis in one assessment, it may occasionally be helpful if no FB is visualized on initial examination.

## Simple Summary

Nasal diseases are common in dogs and cats, and one of the causes includes the presence of nasal foreign bodies (FBs). Clinical signs are not pathognomonic and include nasal discharge, sneezing, epistaxis, and nose pawing. A combination of computed tomography (CT) examination and rhinoscopy is commonly used in the investigation of nasal disease, including cases where FBs are suspected. However, in CT studies, visualization of nasal FBs can be difficult and inconsistent. This present study investigates if displaying CT studies of the head in the lung window (LW) in comparison to the usual bone window (BW) settings could improve the detection rate of nasal FBs.

## 1. Introduction

Diseases of the nasal cavity are common in dogs and cats, and nasal foreign bodies (FBs) have been reported to cause between 1.3% and 18% of nasal diseases in dogs [1,2,3]. Clinical signs are varied including nose pawing, sneezing, nasal discharge, and epistaxis [2,3,4,5], and in chronic cases, the clinical signs are often indistinguishable from other causes of nasal disease, such as neoplasia and inflammatory conditions like lymphocytic–plasmacytic or fungal rhinitis [1,6,7,8,9]. Although dogs of all age and size groups may be affected [5], a previous recent study in the UK reported that this condition is more commonly seen in dogs with a median age at presentation of 3.8 years and heavier than 10 kg [6]. A recent study reported that only 42% of nasal FBs were confidently detected with computed tomography (CT) displaying the images in bone and soft tissue windows in dogs and cats [5]. Metallic or mineralized FBs are easily identified with CT, but small soft tissue attenuating FBs such as vegetal parts especially grass awns can be highly inconspicuous depending on attenuation and size. As reported in the literature, in dogs and cats, nasal FBs can cause secondary changes within the nasal cavity such as focal, mainly unilateral turbinate lysis, accumulation of soft tissue and/or fluid attenuating material, often in the area where the FBs are lodged, together with mucosal thickening [1,2,4,5]. These secondary changes may obscure nasal FBs. In human medicine, the wider display windows, which provide a larger grey scale such as the bone window (BW) and lung window (LW), are commonly used in the investigation of hypoattenuating nasal FBs (for example wooden pieces) with the aim to achieve differentiation from the surrounding air [10,11].

Retrieval of nasal FBs is usually attempted by rhinoscopy; therefore, CT images acquired in high and medium algorithms and displayed in soft tissue window and bone window, may provide helpful supportive information prior to removal regarding location and lateralization of a foreign body (FB), particularly if located in the caudal part of the nasal cavity, and in small breed dogs [1,12].

Even though soft tissue attenuating nasal FBs such as grass awns are particularly challenging to diagnose with CT, in a previous study involving dogs and cats, all visualized grass awns located in airways were only detected when using the LW [13]. Similarly, it was observed that adding the LW in addition to BW facilitates easier diagnosis of some nasal FBs, by subjectively increasing their visualization when compared to the BW. In CT, window settings refer to two key parameters: window width (WW) which determines the range of grey scale value that the image displays, and window level (WL) which defines the centre point of that range. Different window width and window level settings are used to optimize the visibility of specific tissues or structures, such as bone and lung. Images displayed in the BW therefore enhance the visibility of bony structures. On the other hand, images displayed in the LW optimize the visualization of air-filled tissue, emphasizing subtle differences in density where present.

Therefore, it was hypothesized that the use of the LW in addition to BW would increase the diagnostic accuracy for nasal FBs detection [13]. The first aim of this study was to determine if the use of the LW during review of CT studies of the head in dogs results in a higher detection rate of nasal FBs. The second aim was to assess interobserver agreement hypothesizing that there would be a strong interobserver agreement when assessing nasal cavities for the presence of a FB using the LW.

## 2. Material and Methods

### 2.1. Study Population

The present study followed a retrospective, multicentre, observational design. Medical records of two private referral hospitals (Anderson Moores Veterinary Specialists, Winchester, UK; and Davies Veterinary Specialists, Hitchin, UK) were searched for dogs with a rhinoscopically confirmed nasal FB, which underwent CT examination of the head between 2015 and 2023 prior to endoscopy as part of their clinical workup for signs associated with nasal disease. Informed consent was obtained from the owners at the time of admission, granting permission to use clinical data and images for this study.

Dogs were included in the study if they underwent a full CT examination of the head (from the tip of the nose to the occiput) followed by rhinoscopy within 48 h post CT examinations, and if a final rhinoscopic diagnosis was established, confirming and allowing removal of nasal FBs.

Dogs were excluded from the study if FB or FBs were not identified by rhinoscopy, or if the quality of the CT study was not considered diagnostic. As in a previously reported study, cases were also excluded if the FB was located in the nasopharynx only, which represents a different anatomical location [5].

Information gathered from the clinical records included breed, age, gender, clinical history, clinical signs, and duration of the clinical signs. The clinical presentation was considered acute if present for 4 weeks or fewer, and chronic if they were present for more than 4 weeks [5]. Routine hematology and biochemistry results were also recorded if available.

Cases were initially reviewed for inclusion by a 2-year diagnostic imaging resident (N.F.), and the final decision concerning patient inclusion was made by a ECVDI certified veterinary radiologist (P.A.).

### 2.2. Image Acquisition

Computed tomography studies were performed with the patient in sternal recumbency under general anesthesia or under sedation. General anesthesia was induced to effect using up to 1 mg/kg of intravenous Propofol or Alfaxalon and maintained with Isoflurane. Two different 64-slice multidetector CT scanners were used to acquire the studies (SOMATOM Perspective and SOMATOM go. All Siemens Healthineers, Erlangen, Germany). The CT scan parameters included the following: helical acquisition, slice thickness ranging between 0.6 mm and 1.5 mm, 120 and 220 milliamperes, 120 and 130 kilovoltage peak, tube rotation time 0.5 s, matrix size 512 × 512, field of view ranged between 90 and 180 mm, resulting in a pixel size ranging from 0.176 mm to 0.352 mm, and scanning time ranging between 25 s and 60 s. Images were reconstructed using a medium and a high frequency reconstruction algorithm (Head Soft tissue: algorithm—J40s medium, window-mediastinum. Head Bone: algorithm—J80s very sharp, window sinuses).

### 2.3. Rhinoscopy Procedure

The diagnostic rhinoscopy in each case was performed with the animals in general anesthesia, placed in sternal recumbency with the rostral nares angled down, directly by European certified veterinary medicine specialists (ECVIM) or American certified medicine veterinary specialists (ACVIM), or by ECVIM or ACVIM residents under direct supervision of ECVIM or ACVIM certified medicine specialists.

A rigid Karl Storz endoscope (Karl Storz SE & Co, Tuttlingen, Germany) or Pentax flexible bronchoscope (Pentax medical, Tokyo, Japan) were used, depending on animal size and clinician’s judgment. FBs were retrieved via crocodile forceps when visible, or removed via saline nasal flushing.

Biopsy of nasal tissue was performed in 12 cases. Nasal tissue biopsy was obtained through biopsy or crocodile forceps to obtain pieces of tissue, preserved in formalin pot and subsequently processed and examined by a European certified veterinary pathologist (ECVP). Results from histopathological examination were recorded when available.

### 2.4. Image Review

CT images were anonymized and reviewed independently, at different locations, by two board ECVDI-certified veterinary radiologists (with different years of working experience) both of whom were aware that the patients were referred for signs of rhinitis/nasal disease, unaware of the CT findings at the time of review and blinded to the final diagnosis. The images were reviewed individually, and not in consensus, using commercially available Digital Imaging and Communications in Medicine (DICOM) viewer software (OsiriX MD DICOM viewer, Pixmeo, Geneva, Switzerland).

Images in high frequency reconstruction algorithm were displayed using the BW (WL 300 HU, WW 1500 HU) and LW (WL −500 HU, WW 1400 HU), corresponding to presets for these windows in the utilized viewing software and similar to previous studies [5,13]. Adjustments to image WW and WL were made, and images displayed in multiplanar reconstructions (dorsal, sagittal, and transverse) were considered necessary for review of each case. Post-contrast images were not made available for review even when they had been obtained in the original examination based on the findings of a previous study, which showed that post-contrast series did not add any additional information [5]. Assessment of the CT images was based on the methodology of that same study [5].

Evaluators were asked to review the CT images first in the BW and subsequently the same images were displayed in the LW to evaluate the presence or absence of a convincing FB. As described in Moreno-Aguano et al. [5], foreign bodies were defined as abnormal structures located within a nasal meatus or among the nasal turbinates, having a linear or geometric shape clearly differentiable from the normal nasal anatomy. If FBs were not clearly visible, but their presence suspected by the reviewers, this was recorded. The lateralization and approximate location of the nasal FBs were recorded as well as their size (maximal measurement in length, width, and height when possible), shape, homogeneity, and attenuation in Hounsfield Unit (HU).

For this study, the division of the nasal cavity was based on a previous publication [1] that subdivided it into thirds using anatomical landmarks and the modified Triadan dental numbering system [14]. The rostral third extended from the nostril openings to the level of tooth 105 (maxillary first premolar). The middle third extended from tooth 105 to the rostral root of tooth 108 (maxillary fourth premolar). The caudal third began just caudal to the rostral root of tooth 108.

### 2.5. Statistical Analysis

Information on age, gender, breed, duration of clinical signs, and type of FBs were summarized by median and range, or frequency and percentage as appropriate.

Cohen’s kappa (κ values < 0.4 poor agreement, κ value 0.4–0.75 moderate agreement, κ > 0.75 excellent agreement) was calculated to determine interobserver agreement between the two reviewers on the presence or absence of convincing FB in the nasal cavities displayed for each window: bone and lung [15]. McNemar’s test was used for each reviewer to test if detection rates differed between bone and lung windows. Two-way random effects, absolute agreement, single rater intraclass correlations (ICC (2,1)) were used for each window to assess agreement in the maximum dimension recorded by reviewers when both detected a FB, and for each reviewer to assess agreement between the two windows. Definitions of level of agreement for ICC were based on Koo et al., although calculations are based on low numbers [16].

In addition, in instances where the FB was not directly visualized but was suspected to be present, it was coded as ‘suspicious’. Consequently, FB identification was set on an ordinal scale: 2 presence of FB, 1 suspicion of FB and 0 absence of visible FB. Wilcoxon signed-rank tests were used to compare this ordinal scale between bone and lung window for each reviewer separately.

Analysis was undertaken in the R 4.3.3 (R Foundation of Statistical Computing, Vienna, Austria) and Minitab21 (Version 21, Minitab LLC, State College, PA, USA) packages, significance was taken as *p* < 0.05.

## 3. Results

### 3.1. Demographics

A total of 184 dogs were examined between 2015 and 2023 and of these, 47 dogs (25%) met the inclusion criteria, with final diagnosis of endoscopically retrieved nasal FB. The nasal FBs retrieved were 30/47 grass awns (64%), grass blades 8/47 (17%), other plant material 3/47 (6%) and six of other material (e.g., piece of wood, long splinter/mineralized plant material, stone, plastic fragment, grains of sand, stick). In addition, histopathological examination was performed in twelve cases (23%), five cases were diagnosed with neutrophilic rhinitis, four cases with lymphocytic–plasmacytic rhinitis, in two cases *Aspergillus* spp. was isolated, while in one case the samples were not diagnostic. In the remaining cases, histopathology was not performed based on the clinician’s judgment. The decision to perform nasal biopsies for histopathology was made on a case-by-case basis by the medicine specialists, primarily influenced by the changes observed during rhinoscopy and/or according to owners’ financial considerations.

Dogs had a median age of 5 years (interquartile range of 2 to 7 years). There were 30 males (64%), of which 11 were entire (37%) and 19 were castrated (63%); 17 females (36%), of which 2 were entire (12%) and 15 were spayed (88%). Breeds included six Cavalier King Charles Spaniels (14%), four West Highland White Terriers (10%), and three Dachshunds (8%). Two of each of the following breeds included Jack Russell Terriers (4%), Labrador Retrievers (4%), small crossbreeds (4%), Miniature Schnauzers (4%), Cocker Spaniels (4%), Golden Retrievers (4%), Hungarian Vizslas (4%), and Shih Tzus (4%). One of each of eighteen other breeds included a Patterdale Terrier (2%), Welsh Terrier (2%), Australian Shepherd (2%), Sprocker Spaniel (2%), Flat-coated Retriever (2%), Patterdale Terrier cross (2%), Border Terrier (2%), Lurcher (2%), Chihuahua (2%), Working Cocker Spaniel (2%), Basenji (2%), Beagle (2%), Yorkshire Terrier (2%), Basset Hound (2%), Springer Spaniel (2%), Shar Pei (2%), Poodle cross (2%), and Sprocker Spaniel cross (2%).

At the time of presentation, the clinical signs were presented acutely (less than a month) in 20 cases and chronic (over a month) in 27 cases. The most common clinical signs were sneezing in 38 dogs, epistaxis in 17 dogs and nasal discharge in 16 dogs, with most dogs presenting with more than one clinical sign. Findings are summarized in Table 1.

### 3.2. Foreign Body Detection

Each reviewer examined the series of all 47 cases, resulting in a total of 94 reviewed studies. The first reviewer identified 10 FBs out of 47 in the BW, while the second reviewer identified 9 out of 47 FBs in the same window setting. Therefore, a total of 19/94 FBs were confidently detected (equating to an overall sensitivity of 20%) when the studies were displayed in the BW.

In the LW, the first reviewer detected 10 FBs out of 47, and the second reviewer detected 10 out of 47, resulting in a total of 20/94 FBs (equating to an overall sensitivity of 21%) detected when displayed in the LW (Table 2).

The majority of foreign bodies identified by the reviewers were observed in dogs presenting with an acute onset of clinical signs 13/94 (14%), compared to those with chronic signs 7/94 (7%).

The total number of foreign bodies identified by the reviewer is summarized in Table 2.

### 3.3. Statistical Findings

In each window, there was agreement between reviewers on the presence of FBs in six dogs. Of these, two were Cavalier King Charles Spaniels; a neutered female and neutered male; a male entire Hungarian Vizsla; a female neutered Sprocker Spaniel; a male entire Lurcher; and a male entire Dachshund.

The reviewers disagreed in 7 studies when the images were displayed in the BW and 8 studies when the images were displayed in the LW. Therefore, the agreement between reviewers was moderate (κ = 0.53 for BW and κ = 0.49 for LW), indicating weak statistical significance. Detection rates between the two windows did not differ significantly for either reviewer (both *p* = 1.000).

Where both reviewers detected the FB, there was good agreement on maximum dimension (ICC = 0.839 in BW and ICC = 0.852 in LW). Where each reviewer agreed on the presence of a FB in both the BW and LW, there was excellent agreement on maximum dimension (ICC = 0.996 for the first reviewer and ICC = 0.994 for the second reviewer). Furthermore, in these cases, perfect agreement about the nasal cavity in which the FB was located and strong agreement on the attenuation of the FB were demonstrated.

**Figure 1:**
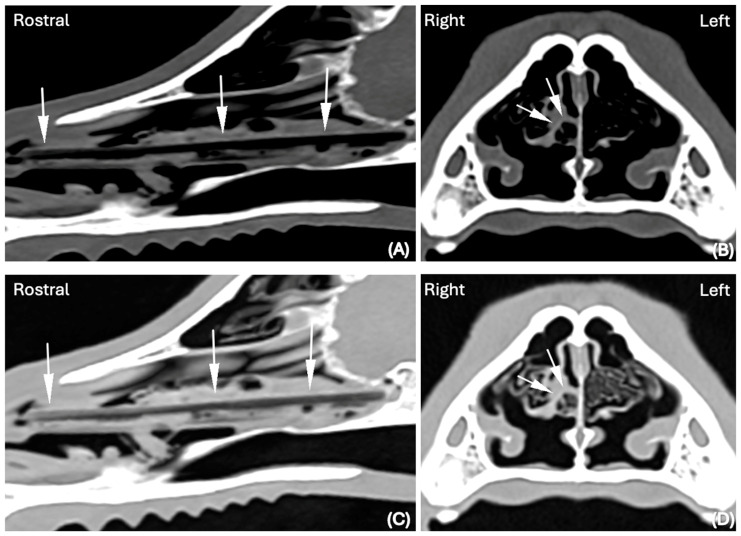
CT images in (**A**) Sagittal and (**B**) Transverse planar reconstruction, displayed in BW (WL 300 HU, WW 1500 HU); scale (DPI 144 × 144). CT images in (**C**) Sagittal and (**D**) Transverse planar reconstruction displayed in LW (WL −500 HU, WW 1400 HU); scale (DPI 144 × 144) of the same dog (Hungarian Vizsla, 2 years old, ME) of the nasal cavity. An elongated, thick hypoattenuating structure was clearly visualized in BW and LW (white arrows) lodged in the right nasal cavity. A long wooden stick was successfully retrieved via rhinoscopy.

In both the LW and BW, the shape of the FBs was elongated in 14 cases (30%), elongated and splitting in two halves in 1 case (2%) and elongated with branching in another case (2%). An example of an elongated nasal FB visible in the BW and LW and detected by both reviewers is visible in Figure 1.

In the remaining cases, the FBs were ellipsoid in appearance or linear with a folded appearance. Fifteen FBs were homogeneous in attenuation, while the remaining were heterogeneous, displaying variable attenuation ranging from −600 HU to 30 HU. The size of the FBs was variable, but in all cases the diameter was highest in the rostro-caudal direction. The location of the nasal FB was very variable throughout the nasal cavity. In the rostro-caudal dimension, most FBs were visible in a rostral and mid-portion of the nasal cavity. The findings are summarized in Table 3.

**Figure 2:**
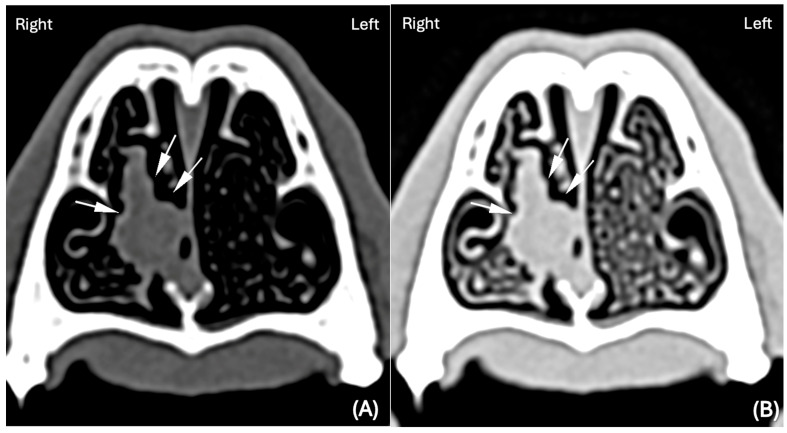
(**A**) Transverse CT image displayed in BW (WL 300 HU, WW 1500 HU); scale (DPI 144 × 144) and (**B**) Transverse CT image displayed in LW (WL −500, WW 1400HU); scale (DPI 144 × 144)) of the rostral third of a canine nasal cavity. The images show unilateral focal moderate accumulation of soft tissue attenuating material (~30–60 HU, white arrows) within the ventromedial portion of the right nasal passage in a dog (Working Cocker Spaniel, 4 years old, MN), surrounded by mild turbinate loss.

In the remaining cases, the FB was not clearly visible. However, some CT abnormalities were present, such as unilateral focal accumulation of fluid to soft tissue attenuating material, with or without associated surrounding mild turbinate loss (Figure 2), that the reviewers considered suggestive of the presence of a FB. In the BW, 30/75 cases (40%) were classified as ‘suspicious’ by the reviewers and 29/74 (39%) were classified as ‘suspicious’ in the LW. Spearman correlations between these ordinal scales were 0.408, *p* = 0.004 in the BW and 0.382, *p* = 0.008 in the LW.

CT abnormalities were detected in the left nasal cavity in 19 cases and in the right nasal cavity in 11 cases. Breeds were different, including three Cavalier King Charles Spaniels, three West Highland White Terriers, two Labrador Retrievers, two small crossbreeds, two Cocker Spaniels, Jack Russell Terriers, Miniature Schnauzers, Border Terriers, Yorkshire Terriers, and one per each of other breeds. There were 13 castrated males, 10 neutered females and 7 entire males. Dogs had an age ranging from 1 year to 13 years.

**Figure 3:**
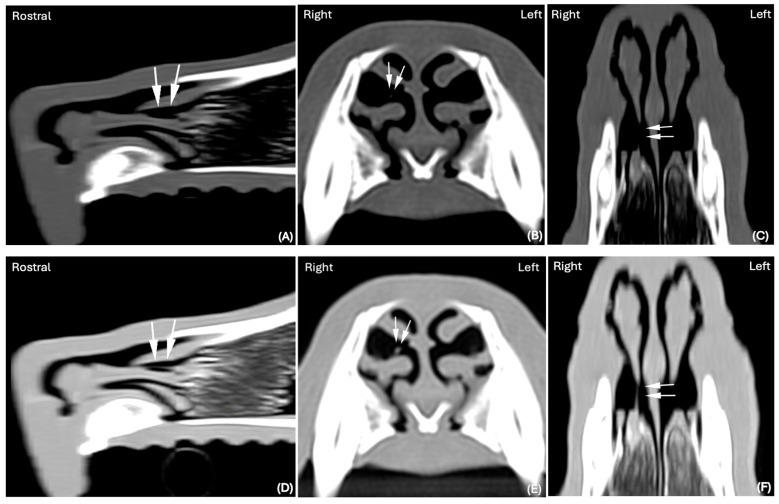
CT images in (**A**) Sagittal, (**B**) Transverse and (**C**) Dorsal planar reconstruction, displayed in BW (WL 300 HU, WW 1500 HU); scale (DPI 144 × 144). CT images in (**D**) Sagittal, (**E**) Transverse, and (**F**) Dorsal planar reconstruction displayed in LW (WL −500 HU, WW 1400 HU); scale (DPI 144 × 144) of the same dog (Labrador, 8 years old, MN), of the rostral third of the nasal cavity. The presence of a nasal foreign body was considered suspicious in BW (white arrows) (**A**–**C**). In the LW, a well-defined thin elongated foreign body was clearly identified (white arrows) (**D**–**F**). A grass blade was successfully retrieved via rhinoscopy.

In one of 30 cases in which the nasal FB was considered suspicious in the BW (Figure 3A–C), it was subsequently confidently detected in the LW (Figure 3D–F).

## 4. Discussion

The results of our study did not support our first hypotheses, as there was no significant improvement in visualization of nasal FBs when using the LW during review of CT images when compared to the BW.

The use of wide window settings (such as BW and LW) in the evaluation of nasal FBs has been previously reported in veterinary medicine [13,17]. The LW is characterized by a lower window level compared with the BW, a feature specifically designed to accentuate air-filled structures and to highlight subtle differences in attenuation, particularly at the interface between soft tissues and air. By enhancing these low-attenuation contrasts, the LW increases the likelihood of detecting non-mineralized or low-attenuating/hypodense foreign material, which may otherwise be obscured within air-filled structures. For example, in human medicine, window settings with a low WL are frequently utilized for the evaluation of hypoattenuating FBs (e.g., wooden pieces) [10,11]. Surprisingly, our findings differ from the results reported in a previous veterinary study, in which plant material (grass seeds) located in the airway was only visible when using a wide WW and low WL, optimized for the assessment of lung parenchyma [13]. We speculate that in the previously reported study, the FBs may have been largely surrounded by air and localized within the bronchial trees, thoracic wall, ear canal, and other locations [13]. Although three FBs in the aforementioned study were described as located in the nasal cavity or nasopharynx, the precise number confined to the nasal cavity alone was not specified. In contrast, all FBs in our study were confined exclusively to the nasal cavity, where the presence of turbinates reduces the effective airspace when compared to the nasopharynx. Consequently, even a relatively small volume of secretion may be sufficient to obscure a nasal FB, limiting the diagnostic utility of the LW in this anatomical region. Furthermore, secretion is likely to accumulate over time, potentially further diminishing the usefulness of the LW in more chronic cases.

As described in material and methods, all the CT scans were acquired using a range of milliamperes, kilovoltage peak, and slice thickness. They were optimized to maximize spatial resolution while maintaining image noise and radiation dose within acceptable level, thereby enhancing the visualization of small structures and/or subtle lesions. Reconstruction algorithms were similarly selected to balance spatial resolution and noise. The fact that the LW did not enhance the diagnostic accuracy for the detection of nasal FBs in our study population is most likely related to the unique anatomical characteristics of the nasal passages, rather than due to limitations of the chosen CT acquisition protocols or reconstruction techniques. These anatomical and physiological differences most probably explain the discrepancies between our results and those reported in the literature, highlighting the importance of tailoring CT window settings to both the anatomical site and the specific characteristics of the FB being evaluated.

Our second hypothesis was also not supported, as the interobserver agreement between reviewers was only moderate. The two reviewers disagreed between cases in which the FB was visualized, disagreeing in seven cases when the images were displayed in the BW (κ = 0.53) and disagreeing in eight cases when the images were displayed in the LW (κ = 0.49). However, in the cases where both observers agreed that a FB was present, there was also perfect agreement about the nasal cavity in which the FB was located and strong agreement about the attenuation of the FB. A possible explanation for the only moderate agreement for presence of a FB is that the studies were reviewed individually, in different geographic locations, without final consensus by the evaluators. It is plausible that collective review would have increased foreign body detection; however, the primary objective of this study was to evaluate independent interobserver agreement. In a previous publication relating to CT diagnosis of nasal foreign bodies, awareness of the final diagnosis by reviewers and CT interpretation in consensus had already been investigated [5]. Furthermore, in clinical practice, consensus review by two radiologists is often not possible at the time a patient is undergoing a CT examination with consecutive rhinoscopy. Therefore, the authors intentionally chose a different approach, in order to avoid introducing a bias and elucidate the ability of CT to detected foreign bodies in the nasal cavity of dogs, in conditions that mirror a common clinical scenario.

Another possible explanation for the moderate interobserver agreement is that the reviewers had the flexibility to adjust the window settings during case evaluations, rather than strictly following the predefined BW and LW parameters. While this lack of standardization may have introduced some variability, it was intentionally allowed to replicate the clinical context, where radiologists routinely perform minor adjustments of the window settings, as well as using the zoom function, and perform multiplanar reconstruction (MPR) to optimize visualization of different structures. Although these adjustments were performed individually by each observer, there was consistency regarding the interpretation protocol, use of the same viewing software (e.g., Osirix MD DICOM viewer) with identical tools, as well as comparable observer proficiency. Restricting evaluation to fixed images would have imposed an artificial limitation, failing to reflect the dynamic nature of CT interpretation. Therefore, while the methodological flexibility in this study may have introduced some variability, it was considered necessary to replicate clinical conditions and provide a valid assessment of interobserver agreement under realistic circumstances.

Detection of nasal FBs is extremely challenging and the difficulty to visualize small plant fragments has been previously reported [1,6,18,19]. In a previous study, only 42% of nasal FBs were confidently detected with CT [5]. When visualized, grass awns can be identified as linear, tubular, or elongated soft tissue to slightly hyperattenuating structures; their visualization is usually improved by the use of MPR [5,13]. However, consistent with previous studies [1,5,6], the vast majority of grass awn FBs in our patients were also not identified (80%) in either the LW or the BW. In addition, reviewers in our study were blinded to the presence of FBs and only had the information that all dogs were referred for rhinitis/nasal disease. Therefore, we can speculate that the presence of secondary changes in the nasal cavity associated with the presence of some FBs in our cases could have not only influenced their visibility, but that it may have also potentially led to satisfaction of search errors. In addition, if the reviewers had been aware of the final diagnosis at the time of review as was the case in the previous study [5], they would have likely classified more FBs as definitely present rather than suspected, thereby increasing the detection rate. Given the low rate of detection of nasal FBs, and the only moderate interobserver agreement, it may be advisable to review CT examinations of patients presented for nasal disease routinely by more than one reviewer.

The size and the shape of the FBs could also play a role in their detection. Grass awns can have a low diameter in short axis similar to the surrounding nasal turbinates and a thin linear shape, which may make them inconspicuous. We also speculate that not only the size of FBs might impact FB detection, but also the skull conformation of the patient, as reduced space between the nasal turbinates might interfere in the identification of FBs. However, further studies are required since the skull conformation was not evaluated in this study.

Although patient weight was not specifically recorded in our study, the majority of dogs included in this study were small- to medium-sized dog breeds such as terriers and spaniels. This is similar to other recent published data in the UK [5,6] indicating a potential predisposition in these breeds, due to strong hunting instincts and a tendency for exploratory activity.

In one case where the FB was considered “suspicious” in the BW, it was confidently detected when displayed in the LW, showing that using the latter might have achieved better delineation of its margins, as surrounded by air, and therefore its visualization in this isolated case (Figure 2).

The potential value of alternative imaging modalities warrants consideration. Where available, magnetic resonance imaging (MRI) may serve as a complementary or even initial diagnostic tool for the evaluation of the nasal cavities. Magnetic resonance imaging (MRI) has been shown to allow detection of foreign bodies in dogs, for example, in the case of oropharyngeal injuries [20]. However, CT has several advantages compared to MRI in the diagnosis of nasal foreign bodies, including greater spatial resolution, absence of magnetic susceptibility artifacts associated with gas-filled structures, as well as easier volumetric acquisition, facilitating easier three-dimensional reconstructions and generally faster acquisition times [21,22,23]. Relevant literature specifically assessing the ability of MRI to detect foreign bodies in dogs remains limited, consisting of case reports or case series. These publications generally do not focus on evaluation of the nasal cavity or provide direct comparison between MRI and CT in live subjects. In a previous cadaveric investigation, CT was found to be superior to both MRI and ultrasonography for the detection of foreign bodies in the canine manus [23]. Ultimately, future studies comparing CT and MRI to rhinoscopy as a gold standard would be required to establish meaningful conclusions. In settings where CT or MRI examinations are not available or financially prohibitive, rhinoscopy could also be considered as a first line modality. However, CT and rhinoscopy may provide complementary information so that CT examination followed by rhinoscopy offers a more comprehensive and reliable diagnostic pathway, and this approach is therefore also most commonly used in clinical practice.

## 5. Limitation and Future Recommendation

There were several limitations to this study. Firstly, the small sample size may have led to a Type II error. A Type II error refers to the failure to reject a false null hypothesis, resulting in a false negative finding. In the context of this study, a Type II error would imply that the use of the LW is indeed associated with improved diagnostic accuracy for the detection of nasal foreign bodies, but that this association was not identified in the analysis. It remains uncertain whether including a larger number of cases would have resulted in a statistical significance between the two display windows. Secondly, case selection may have made a difference. While including both acute and chronic cases, the majority of the dogs in our study had a duration of the clinical signs of more than 4 weeks. Chronic cases usually display more severe changes in the nasal cavities such as fluid accumulation, cellular content, turbinate lysis, and mucosal thickening [2], and small vegetable parts might become partially lysed or effaced by the surrounding accumulation of material, obliterating and therefore limiting the visibility of the FB as described above. Indeed, the majority of the foreign bodies identified by the reviewers were detected in dogs presenting with an acute onset of clinical signs, compared to chronic cases.

Thirdly, as mentioned in the material and methods, the reviewers were blinded to the presence or absence of a FB and only knew all dogs were referred for rhinitis. The reviewers were asked to evaluate the cases first in the BW and then in the LW, filling out one questionnaire for each presented window per case. It is possible that assessing the images first in the BW might have biased the reviewers in the consecutive assessment of the images in the LW, and this could have influenced the results.

As mentioned above, another limitation of this study is that the reviewers did not review the studies in consensus. A subsequent combined review of the cases, particularly those in which disagreements occurred, or those where signs suggestive of foreign body rhinitis were identified, and foreign body suspected, could potentially have changed their assessments when scoring the cases. In addition, the reviewers were allowed to freely adjust the window settings when reviewing the case. This lack of standardization could have introduced interpretative variability between reviewers. While the authors acknowledge these limitations, the aim of this study was to replicate conditions representative of clinical imaging practice.

Based on our literature review, this is the first study which assessed interobserver agreement in the diagnosis of nasal FBs, and it may be helpful to examine in future studies whether consensus review increases detection rate.

## 6. Conclusions

In conclusion, our findings indicated that the use of the LW does not significantly increase the detection rate of nasal FBs when compared to the BW. However, shifting between the BW and LW might potentially help the identification and evaluation of hypoattenuating nasal FBs in isolated cases, particularly with small fragments of plant material which are not surrounded by a large amount of secretion.

## Figures and Tables

**Table 1:** Onset of clinical presentation and most common clinical signs categorized by breed sex and neuter status (male neutered MN, male entire ME, female neutered FN and female entire FE).

Onset of Presentation	Most Common Clinical Signs *	Breed	Sex
Acute 20/47 (42%)	15 Sneezing 6 Epistaxis 3 Nasal Discharge	Hungarian Vizsla (2), Labrador Retriever (2), Jack Russell Terrier (1), Miniature Schnauzer (1), Dachshund (1), Patterdale Terrier (1), West Highland White Terrier (1), Small crossbreed (1), Lurcher (1), Chihuahua (1), Working Cocker Spaniel (1), Beagle (1), Basset Hound (1), Cavalier King Charles Spaniel (1), Golden Retriever (1), Poodle cross (1), Sprocker cross (1), Cocker Spaniel (1).	9 MN 6 ME 5 FN 0 FE
Chronic 27/47 (58%)	23 Sneezing 13 Nasal Discharge 11 Epistaxis	Cavalier King Charles Spaniel (5), West Highland White Terrier (3), Dachshund (2), Shih Tzu (2), Miniature Schnauzer (1), Golden Retriever (1), Jack Russell Terrier (1), Welsh Terrier (1), Australian Shepherd (1), Sprocker Spaniel (1), Flat-coated Retriever (1), Patterdale Terrier Cross (1), Border Terrier (1), Basenji (1), Yorkshire Terrier (1), Cocker Spaniel (1), Springer Spaniel (1), Small crossbreed (1), Shar pei (1)	10 MN 5 ME 10 FN 2 FE

* Most patients were presented for more than one clinical sign.

**Table 2:** Overall detection rate of the foreign bodies (FBs) in bone window and lung window.

Window Settings	Reviewers	FBs Detected (Out of 47 Cases)	Overall Detection Rate
Bone Window	Reviewer 1	10	–
Bone Window	Reviewer 2	9	–
Bone Window	Combined	19	(20%)
Lung Window	Reviewer 1	10	–
Lung Window	Reviewer 2	10	–
Lung window	Combined	20	(21%)

**Table 3:** Shape, dimension, attenuation of the foreign bodies (FBs) in bone window (BW) and lung window (LW), by localization and side of the nasal cavity and breed in which they were identified.

Breed	Side	Location	Shape of the FB	Dimensions Length × Width × Height in mm	Attenuation in BW	Attenuation in LW
Cavalier King Charles Spaniel	Right	Rostral	Elongated	35 × 1.5 × 1.5	Homogeneous	Homogeneous
Hungarian Vizsla	Right	Rostral	Elongated	4 × 4 × 90	Heterogeneous	Heterogeneous
Dachshund	Left	Rostral	Elongated	6 × 2 × 2	Homogeneous	Homogeneous
Cavalier King Charles Spaniel	Right	Mid	Elongated	6 × 2 × 2	Heterogeneous	Heterogeneous
Welsh Terrier	Right	Caudal	Elongated	6.6 × 1 × 1	Heterogeneous	Heterogeneous
Sprocker Spaniel	Right	Mid	Elongated	42 × 1 × 1	Homogeneous	Homogeneous
Dachshund	Right	Choana	Elongated with branches	30 × 1 × 1	Homogeneous	Homogeneous
Patterdale Terrier cross	Left	Caudal and Ventral	Ellipsoid	2 ×1 × 1	Homogeneous	Homogeneous
Hungarian Vizsla	Right	Mid and ventral	Elongated geometric	85 × 4.4 × 5	Homogeneous	Homogeneous
Lurcher	Right	Caudal and Ventral	Elongated	7 × 2 × 2	Homogeneous	Homogeneous
Working Cocker Spaniel	Right	Mid and Ventral	Elongated	15 × 3 × 3	Heterogeneous	Heterogeneous
Dachshund	Left	Caudal and Ventral	Elongated	54 × 3 × 3	Homogeneous	Homogeneous
Cavalier King Charles Spaniel	Right	Mid	Elongated	10 × 2 × 3.3	Homogeneous	Homogeneous
Sprocker Spaniel	Right	Mid and ventral	Elongated	50 × 2.2 × 2.9	Homogeneous	Homogeneous
Labrador Setriever	Left	Ventral	Elongated	20 × 1.8 × 1.8	Homogeneous	Homogeneous
Small crossbreed	Left	Rostral and Dorsal	Elongated	13 × 0.8 × 0.7	Homogeneous	Homogeneous
Lurcher	Right	Ventral	Linear, folded	45 × 2 × 1	Homogeneous	Homogeneous
Dachshund	Left	Caudal	Elongated	60 × 1.2 × 1.2	Homogeneous	Homogeneous
Springer spaniel	Right	Rostral	Elongated, “Arrow shape”	8 × 0.8 × 1	Homogeneous	Homogeneous
Labrador	Right	Rostral	Elongated	18 × 1 × 1	_	Homogeneous

## Data Availability

The original contributions presented in this study are included in the article. Further inquiries can be directed to the corresponding author.

## Abbreviations

ACVIM	American College of Veterinary Internal Medicine
BW	Bone Window
CT	Computed Tomography
DICOM	Digital Imaging and Communication in Medicine
ECVDI	European College of Veterinary Diagnostic Imaging
ECVIM	European College of Veterinary Internal Medicine
ECVP	European College of Veterinary Pathologists
FB	Foreign body
FBs	Foreign bodies
FE	Female entire
FN	Female neutered
HU	Hounsfield Unit
LW	Lung Window
ME	Male entire
MN	Male neutered
MPR	Multiplanar Reconstruction
MRI	Magnetic Resonance Imaging
WL	Window Level
WW	Window Width
